# Dynamic Inter-Brain Networks Correspond With Specific Communication Behaviors: Using Functional Near-Infrared Spectroscopy Hyperscanning During Creative and Non-creative Communication

**DOI:** 10.3389/fnhum.2022.907332

**Published:** 2022-06-02

**Authors:** Xinyue Wang, Yu Zhang, Yingyao He, Kelong Lu, Ning Hao

**Affiliations:** Shanghai Key Laboratory of Mental Health and Psychological Crisis Intervention, School of Psychology and Cognitive Science, East China Normal University, Shanghai, China

**Keywords:** inter-brain synchrony, dynamic inter-brain networks, creative communication, hyperscanning, fNIRS (functional near-infrared spectroscopy)

## Abstract

Social interaction is a dynamic and variable process. However, most hyperscanning studies implicitly assume that inter-brain synchrony (IBS) is constant and rarely investigate the temporal variability of the multi-brain networks. In this study, we used sliding windows and k-mean clustering to obtain a set of representative inter-brain network states during different group communication tasks. By calculating the network parameters and temporal occurrence of the inter-brain states, we found that dense efficient interbrain states and sparse inefficient interbrain states appeared alternately and periodically, and the occurrence of efficient interbrain states was positively correlated with collaborative behaviors and group performance. Moreover, compared to common communication, the occurrence of efficient interbrain states and state transitions were significantly higher during creative communication, indicating a more active and intertwined neural network. These findings may indicate that there is a close correspondence between inter-brain network states and social behaviors, contributing to the flourishing literature on group communication.

## Introduction

Humans are inherently social animals with a natural desire to communicate. Therefore, many researchers in the field of human neuroscience have been making every effort to understand the multi-brain neural mechanisms of social communication. In recent years, hyperscanning technology has attracted lots of attention and has been acclaimed as a “game changer” in social interaction studies (Gvirts and Perlmutter, 2020). Hyperscanning is a measurement that records brain activity of two or more individuals at the same time (Montague et al., 2002; Li et al., 2009). By using hyperscanning, researchers can measure the strength of inter-brain neural coupling (termed inter-brain synchrony, IBS), which provides a tool for revealing the basis of multi-brain neural mechanisms behind complex social activities.

Previous hyperscanning studies showed that inter-brain synchrony (IBS) arises when individuals communicate with each other, inferring others’ intentions, and cooperate to achieve common goals (Cui et al., 2012; Jiang et al., 2012; Nozawa et al., 2016). For instance, Cui et al. (2012) measured brain activity of paired participants when they were performing two different tasks: a cooperation task where the participants try to press the button at the same time, and a competition task where the participants try to press the button as fast as possible. Results showed significantly higher IBS in the superior frontal cortex under the cooperation task than in the competition task. Lu and Hao (2019) measured brain activity of three-person groups where two of the members are real participants and one is a confederate, and found that IBS between the real participant pairings is significantly higher than the pairs between real participants and confederates. In addition, researchers also found that IBS emerges during natural social communication (Nozawa et al., 2016). Based on these results, IBS has been seen as an effective neural mark of inter-brain information transmission and shared intentionality (Fishburn et al., 2018).

Social communication is a complex and dynamic system. During social interaction, people constantly update information and adjust communication strategies, and their inter-brain neural networks change accordingly. However, most hyperscanning studies implicitly assume that IBS is steady throughout the entire recording procedure and rarely investigate the temporal variability of the multi-brain networks. Recently, Li et al. (2021) presented a novel approach based on sliding windows and k-mean clustering to capture the dynamic modulation of IBS patterns during cooperation tasks and found different IBS states occurred at different stages of the task. Here we followed their methods using sliding windows and k-mean clustering to characterize dynamic IBS (dIBS) states during group communication and applied multiple behavioral indices to explore the connection between different dIBS states and social behaviors. By calculating the graph-based network parameters and temporal occurrence of the dIBS states, as well as associating them with behavioral indices, we analyzed the variability and flexibility of the dynamic inter-brain networks during different social communication tasks.

Creative communication (i.e., communication and brainstorming in creative collaborations) is a special type of communication. It involves a distinct constellation of communication challenges, such as sharing generated ideas, understanding and evaluating others’ ideas, and integrating personal novelty into collaborative work (Jordan and Babrow, 2013); all in order to produce novel and applicable products (Vera and Crossan, 2005; Paulus and Brown, 2007; Runco and Jaeger, 2012). It is an indispensable driving force for the development of modern society. Therefore, in this study, we implemented two different tasks to explore the similarities and differences of the dIBS states between creative and non-creative group communication tasks.

**FIGURE 1:**
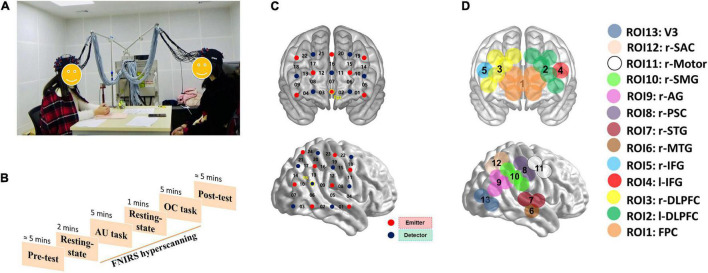
The experimental settings of the current study. **(A)** Two participants were communicating with each other during the tasks. “AU task” means alternative uses task; “OC task” means object characteristic task. **(B)** The experiment procedure. **(C)** The optode probe sets. **(D)** Regions of interest.

Previous studies have found that IBS can occur in multiple brain regions, such as the prefrontal cortex (PFC), right temporal-parietal junction (r-TPJ), superior temporal gyrus (STG), and medial temporal gyrus (MTG). These areas are deeply involved in production and comprehension of conversation, “reading” other minds, and anticipating their future actions, all of which contributed to social communication (Rizzolatti and Craighero, 2004; Frith and Frith, 2007). For instance, the PFC are important to tasks involving cooperation and social interactions (Decety et al., 2004; Cui et al., 2012; Baker et al., 2016). IBS in this area may be related to inferring others’ goals and intentions (Stephens et al., 2010; Silbert et al., 2014). Previous studies also found that the r-TPJ is associated with understanding meanings of the conversation, and the increased IBS in this area was observed during group collaborations and storytelling (Jung-Beeman, 2005; Redcay et al., 2010; Hari et al., 2015). Besides, in some sensory or motor-related areas, IBS can also be induced by shared external stimulus (Hasson and Frith, 2016). Accordingly, these areas were selected as regions of interest in the present study (see details in Figure 1D) to explore the dynamic inter-brain networks during group communication.

Compared to functional magnetic resonance imaging (fMRI) and electroencephalography (EEG), functional near-infrared spectroscopy (fNIRS) has its unique advantages. During the recording procedure, fNIRS allows participants to perform small movements and has great adaptability to various environments, which benefits to recording brain activities in natural social scenes. Therefore, many researchers applied fNIRS-based hyperscanning technology to explore multi-brain neural interactions during real-life social communication (Jiang et al., 2012; Lu et al., 2019). In the present study, we also used fNIRS-based hyperscanning to track brain activity in pairs of participants during group communication tasks. Fifty-four individuals were randomly assigned as 27 dyads and were required to complete a creative communication task (alternative uses task; AUT) and a common communication task (object characteristic task; OCT). We adopted wavelet transform coherence, sliding windows, and k-mean clustering to describe participants’ dIBS states. By calculating the graph-based network parameters and temporal occurrence of the dIBS states, as well as associating them with behavioral indicators, we analyzed the variability of the inter-brain networks during different group communication. We hypothesized that in both communication tasks, different dIBS states will appear alternately and periodically, and the occurrence of dense efficient dIBS states will be associated with better group communication performance. Moreover, considering that creative communication has its distinct social challenges, we hypothesized that compared to the non-creative communication, the inter-brain network will be more flexible during the creative communication.

## Materials and Methods

### Participants

Fifty-four participants (all female, age: 21.2 ± 2.0 years) were randomly assigned as 27 dyads. Participants were recruited through online advertising and were each paid ¥40 for their participation. Before the experiment, informed consent was provided. The study procedure was approved by the University Committee on Human Research Protection of East China Normal University.

### Procedures

This study consisted of a one factorial design (Task: AUT vs. OCT), with Task as the within-subject factor. The sequence of the AUT and OCT was counterbalanced. The experimental procedure consisted of a 2-min rest session, two 5-min task sessions, and another 1-min rest session (see details in Figure 1). Before each task session, the rules of communication and task instructions were presented. During the AUT session, participants were asked to report as many unusual and original uses as possible for an ordinary item. During the OCT session, participants were asked to generate as many relevant characteristics as possible of a common item. During both communication tasks, participants were asked to take turns to report, one idea at a time. If a participant cannot think of any idea during their turn, they can say “pass” and present the idea in the next turn.

### Pre- and Post-experimental Tests

Prior to the experiment, participants were asked to complete the self-assessment manikin scale to rate the valence and arousal of their emotional states (Bradley and Lang, 1994; Kirsch et al., 2005; Coan et al., 2006). Immediately after the experiment, participants were asked to rate their emotional state again. They then rated feelings of difficulty, depletion, and enjoyment when performing each task on scales ranging from 1 (“not at all”) to 5 (“very much”).

### Behavioral Assessments

Participants’ performance on the AUT was measured from three dimensions: fluency, originality, and flexibility (Guilford, 1967). The fluency score was the total number of non-redundant responses. The originality score was accessed using an objective method. If a response was statistically infrequent (i.e., 5% or fewer participants in the sample presented the response), it scored 1, and all the other frequent responses (i.e., 95% or more participants in the sample presented the response) scored zero. The originality score was the total number of statistically infrequent responses. The flexibility score was coded according to the number of categories of generated responses (e.g., decorations, weapons, toys, etc.). To compensate for the effect of fluency, the final flexibility score was defined as the number of categories divided by the fluency. Two trained raters independently assessed the originality and flexibility scores for each participant. The inter-rater agreements of originality [internal consistency coefficient (ICC) = 0.89, calculated as Cronbach’s α] and flexibility (ICC = 0.82) were satisfactory. The ratings of the two raters were averaged. The score for each dyad was the sum of the two members.

Participants’ performance on the OCT was scored by fluency, and the scoring procedure was the same as the AUT.

The index of cooperation (IOC) was calculated based on the number of combined ideas, which reflects the perspective-taking behaviors (Larey and Paulus, 1999). First, the responses of the two participants were listed in chronological order. From the first idea to the last, when a response pertained to the same category as the previous response, it scored “1.” The total number of responses scored “1” was defined as the “converge.” The IOC value for each dyad was then obtained using the following equation: IOC = converge/(group fluency—converge). Therefore, this index could demonstrate the extent to which the group members combined their ideas with others, and reveal the level of cooperation between group members (Lu et al., 2019, 2022).

### Functional Near-Infrared Spectroscopy Data Acquisition and Pre-processing

The concentrations of oxyhemoglobin (HbO) and deoxyhemoglobin (HbR) were recorded continuously using an ETG-7100 NIRS system (Hitachi Medical Corporation) for each dyad. Based on the abovementioned studies showing the important contributions of the PFC and right temporal-parietal-occipital regions (r-TPO) to social communication, we placed two optode probe sets on each participant’s PFC (3*5 optode probe set; 22 measurement channels) and r-TPO areas (4*4 optode probe set; 24 measurement channels). The registration of the probe sets was based on the 10–20 system of EEG. The MNI coordinates of the CHs in a typical participant are presented in Supplementary Table 1.

Considering that the HbO signal is more sensitive to changes in cerebral blood flow than the HbR signal, we focused on the HbO signal (Hoshi, 2007; Jiang et al., 2012). The data were pre-processed using a principal component spatial filter algorithm to eliminate the effects of systemic components such as blood pressure, respiratory variation, and blood flow variation on the fNIRS data (Zhang et al., 2016). We also used a correlation-based signal improvement method to remove motion artifacts (Cui et al., 2010). Besides, the initial and last 30-s periods of each task session were removed to obtain data within the steady period, leaving a total of the 480-s period for two task sessions.

### Functional Near-Infrared Spectroscopy Data Analysis

The dynamic IBS analysis was conducted by three processes: (1) IBS computation using wavelet transform coherence, (2) temporal segmentation using sliding windows, and (3) characterization of dIBS states using k-means clustering (Li et al., 2021).

#### Inter-Brain Synchrony Computation

We used wavelet transform coherence (WTC) to assess the cross-correlation (i.e., IBS) between two HbO time series (Grinsted et al., 2004). Fisher’s *r*-to-*z* transformation was applied to IBS values before further analysis (Cui et al., 2012; Simony et al., 2016). In each dyad, we calculated the IBS of all ROI combinations between two participants. The IBS between the same ROI pairings was then averaged. For example, the IBS between IFG-1 (participant 1’s IFG) and AG-2 (participant 2’ AG) was averaged with the IBS between AG-1 (participant 1’AG) and IFG-2 (participant 2’ IFG) (Li et al., 2021), which led to a total of 91 ROI combinations per dyad.

To identify the frequency band of interest (FOI) in this study, we conducted paired sample *t*-tests to compare IBS between the task period and rest period of each ROI combination along the full frequency range (0.01–0.7 Hz). The IBS was averaged across the different task periods prior to the aforementioned *t*-test to prevent bias (Lu et al., 2019; Pan et al., 2020). Data over 0.7 Hz were not taken into account to exclude higher frequency noise, such as cardiac activity (0.8–2.5 Hz). Data below 0.01 Hz were also excluded to avoid fluctuations at very low frequencies (Barrett et al., 2010). The *t*-test results were thresholded at *P* < 0.000005. No further corrections were applied since this analysis was only used to identify the FOI rather than to obtain the final results (Dai et al., 2018; Zheng et al., 2018). Significant *P*-values which survived the thresholding were observed at frequencies between 0.10 and 0.19 Hz (corresponding to the period between 5.2 and 10.5 s), which means in this frequency band, the IBS of the task period was significantly higher than that of the rest period. Therefore, this band was selected as the FOI, and the IBS of each ROI combination was averaged across the selected FOI.

#### Clustering Analysis (Sliding Windows and k-Means Clustering)

**FIGURE 2:**
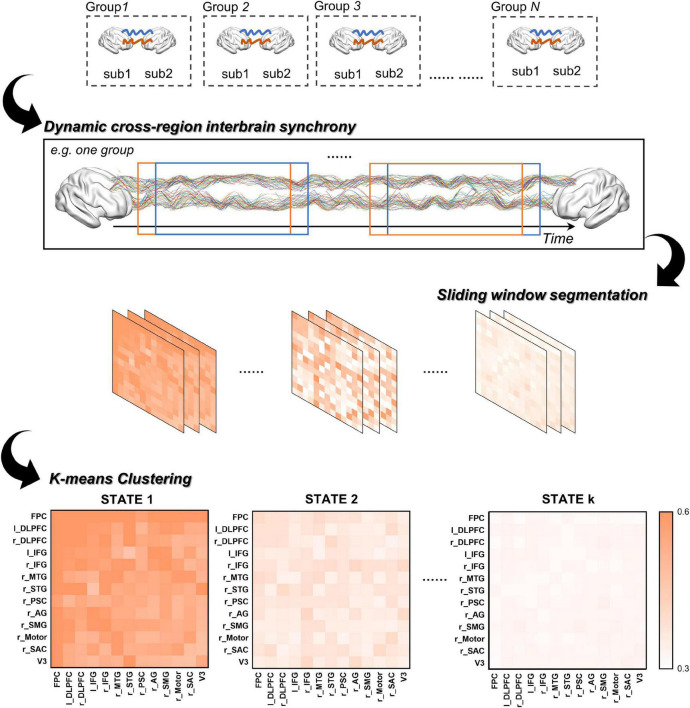
The procedure of clustering analysis.

To quantitively compare the dIBS states between different group communication, the dynamic IBS network of the AUT and OCT were clustered together. First, we used a sliding window approach to obtain a series of windowed IBS matrices along the 480 s task period (see details in Figure 2). The window size was set to 10 s and moved in an increment of 1 s throughout the task. The IBS values were then averaged between the same ROI combinations within each time window to obtain the corresponding IBS matrix. The 480 s task duration was then split into a series of windowed IBS matrices (13 ROIs × 13 ROIs × 471 windows) for each dyad.

**FIGURE 3:**
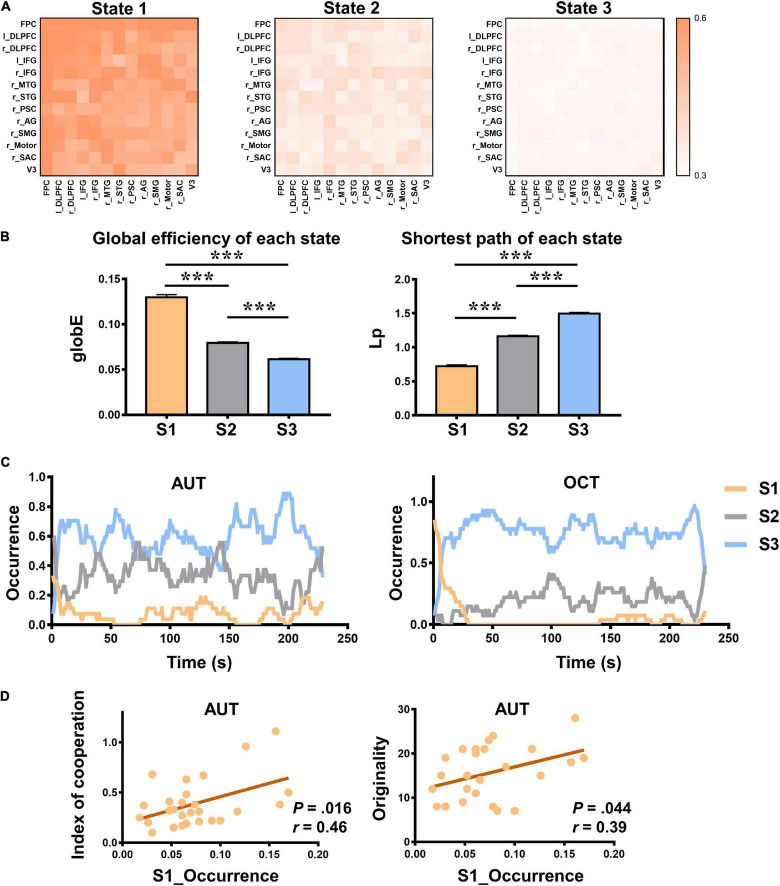
The properties of dynamic inter-brain synchrony (dIBS) states. **(A)** The 13 × 13 matrix of dIBS states. The horizontal and vertical coordinates are ROIs. Color represents the IBS value. **(B)** The global efficiency (globE) and shortest path (Lp) of each dIBS state. Error bars indicate standard errors of the mean. ***P < 0.001. **(C)** The temporal occurrence of each dIBS state during the AUT and OCT. **(D)** Pearson correlations between the occurrence rate of State 1 and the index of cooperation as well as AUT originality. S1 means State 1; S2 means State 2; S3 means State 3.

Next, we averaged these chained IBS matrices across groups and applied a k-means clustering method in MATLAB to assess the similarity between the windowed IBS matrices and obtain the representative dIBS states (i.e., clusters). We chose the number of clusters using the elbow criterion of the cluster validity index, which is the ratio between within-cluster distance to between-cluster distance (Allen et al., 2014; Fang et al., 2020; Li et al., 2021). Specifically, the validity index for different k values were computed and plotted as a function of cluster number, then the number of clusters is chosen at the elbow of the curve to best balance the cost of clustering (i.e., minimize the within-cluster distance and maximize the between-cluster distance) and the number of clusters. In addition, previous research found that the Manhattan distance is a more effective similarity measure than the Euclidean distance for high-dimensional neuroimaging data (Aggarwal et al., 2001), therefore we used the Manhattan distance to calculate the similarity between the windowed IBS matrices. Finally, after iterated 1,000 times to decrease the chances of escaping local minima, the cluster centroids (i.e., the representative dIBS states in Figure 3A) were obtained. These cluster centroids derived from the group-averaged IBS matrices were then used as the initial centroids for the cluster analysis of individual dyad to obtain the final dIBS states of each dyad.

### Statistical Analysis

The dIBS states were characterized by the following metrics: the occurrence rate of each state, number of transitions between states, and network parameters of each state (Allen et al., 2014; Li et al., 2021). The occurrence rate of each state means the percentage of total windows each dIBS states account for. The number of transitions stands for the total number of switches between any two states. Moreover, we also implemented network analyses in MATLAB using GRETNA to calculated graph-based metrics, such as global efficiency (globE) and shortest path length (Lp), of each dIBS state (Achard and Bullmore, 2007; He et al., 2009; Wang et al., 2015). globE is an important network parameter that measures the global efficiency of parallel information transfer in the network. It is the inverse of the harmonic mean of the characteristic path length between each pair of nodes within the network. As for Lp, it quantifies the ability for information propagation in parallel over the whole network, computed as a harmonic mean length between all pairs of nodes. Repeated measure ANOVA was used to assess whether there were significant differences between the characteristics of these dIBS states. Repeated measure ANOVA was also used to assess whether there were significant differences between AUT and OCT in terms of the occurrence rate of three dIBS states and the number of state transitions. In addition, we also examined the Pearson correlation between the properties of each dIBS state and the behavioral group communication performance.

## Results

### Dynamic Interbrain Synchrony States in Group Communication

To quantitively compare the dynamic inter-brain networks between different group communication tasks, the dIBS of the AUT and OCT were clustered together. During the entire group communication, three distinct dIBS states were obtained using the k-means clustering analysis (see details in Figure 3A). Then we calculated graph-based network parameters such as globE and Lp of each state in MATLAB using GRETNA (Achard and Bullmore, 2007; He et al., 2009; Wang et al., 2015). Repeated measure ANOVA was used to assess whether there were significant differences between these dIBS states. Results showed that State 1 had significantly higher globE and significantly lower Lp than State 2 and State 3, and State 2 had significantly higher globE and significantly lower Lp than State 3 [see details in Figure 3B; globE: *F*_(2, 26)_ = 328.47, *P* < 0.001, η*_*p*_^2^* = 0.96, *M_*s*1_* = 0.13, *M_*s*2_* = 0.08, *M_*s*3_* = 0.06; Lp: *F*_(2, 26)_ = 588.16, *P* < 0.001, η*_*p*_^2^* = 0.98, *M_*s*1_* = 0.72, *M_*s*2_* = 1.16, *M_*s*3_* = 1.50; *post hoc* Bonferroni correction was used to account for multiple comparisons].

Repeated measure ANOVA was also used to assess whether there were significant differences between the occurrence rate of these dIBS states. Results showed that in both AUT and OCT, State 1 occurs significantly less than States 2 and 3, and State 2 occurs significantly less than State 3 [AUT: *F*_(2, 26)_ = 261.64, *P* < 0.001, η*_*p*_*^2^ = 0.95, *M_*s*1_* = 0.08, *M_*s*2_* = 0.33, *M_*s*3_* = 0.59; OCT: *F*_(2, 26)_ = 482.66, *P* < 0.001, η*_*p*_^2^* = 0.97, *M_*s*1_* = 0.06, *M_*s*2_* = 0.19, *M_*s*3_* = 0.75; *post hoc* Bonferroni corrected]. It may indicate the difficulty of entering into a state where group members experiencing efficient interbrain information transmission during both creative and non-creative social communication.

Moreover, in Figure 3C, we displayed the temporal occurrence of each dIBS state. Intuitively, it can be seen that different states appeared periodically and alternately during both creative and non-creative communication. We also examined the Pearson correlation between the occurrence rate of each dIBS state and the collaborative behaviors as well as the group performance. Results showed that the occurrence rate of State 1 was positively correlated with group originality and index of cooperation (see details in Figure 3D; AUT originality: *r* = 0.39, *P* = 0.044; IOC: *r* = 0.46, *P* = 0.016).

### Differences Between the Creative Communication and Non-creative Communication

**FIGURE 4:**
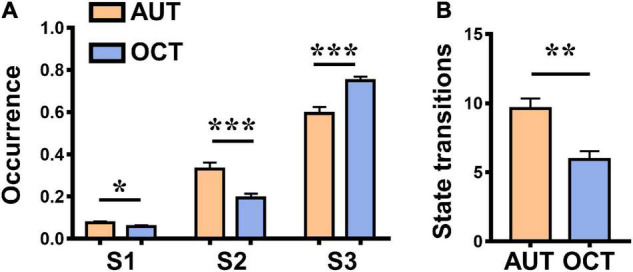
Differences between the alternative uses task (AUT, creative communication) and object characteristic task (OCT, non-creative communication). **(A)** Differences between the occurrence rate of three dIBS states in the AUT and OCT. **(B)** Differences between the state transitions in the AUT and OCT. Error bars indicate standard errors of the mean. *P < 0.05, **P < 0.01, and ***P < 0.001.

Repeated measure ANOVA was used to assess whether there were significant differences between the occurrence rate of three dIBS states in the AUT and OCT. Results showed that during the AUT, the occurrence rate of States 1 and 2 was significantly higher than during the OCT [see details in Figure 4A; State 1: *F*_(1, 26)_ = 4.90, *P* = 0.036, η*_*p*_^2^* = 0.16, *M*_*AUT*_ = 0.08, *M*_*OCT*_ = 0.06; State 2: *F*_(1, 26)_ = 15.71, *P* = 0.001, η*_*p*_^2^* = 0.38, *M*_*AUT*_ = 0.33, *M*_*OCT*_ = 0.19), and the occurrence rate of State 3 was significantly lower than during the OCT [State 3: *F*_(1, 26)_ = 19.45, *P* < 0.001, η*_*p*_^2^* = 0.43, *M*_*AUT*_ = 0.59, *M*_*OCT*_ = 0.75]. In addition, we also measured the number of state transitions during each group communication task and found that the state transitions were significantly higher in the creative communication than in the non-creative communication [see details in Figure 4B; *F*_(1, 26)_ = 13.64, *P* = 0.001, η*_*p*_^2^* = 0.34, *M*_*AUT*_ = 9.59, *M*_*OCT*_ = 5.93]. These findings suggest that compared to the non-creative communication task, participants exhibited a more efficient and flexible interbrain neural network when performing the creative communication task.

### Validation Analysis

We conducted a validation test using pseudogroups (i.e., randomly rearranging the participants to form sham dyads). All analyses were applied to the pseudodata in the same manner as for the empirical data. Specifically, we calculated the dIBS matrices of the 27 pseudodyads (the sample size was the same as that of real participants) and obtained 3 representative dIBS states using k-means clustering. Then we calculated graph-based network parameters such as globE of each state in MATLAB using GRETNA (Achard and Bullmore, 2007). Repeated-measures ANOVA was used to assess whether there were significant differences between the globE of these dIBS states. This permutation process was repeated 300 times. Different from real condition, over 95% pseudogroups showed no significant difference between the globE of three dIBS states, and also no significant difference between the occurrence rate of State 1, State 2, and State 3. Moreover, we also examined the observed positive correlation between communication behaviors and the occurrence rate of dIBS states. Considering it was difficult to calculated the IOC scores of pseudodyads, we only examined the correlation between AUT originality and the occurrence rate of dIBS states. The originality score of the pseudodyads were the sum of the two members. Different from real condition, over 95% of pseudogroups showed no significant correlations between AUT originality and the occurrence rate of any dIBS states. This may indicate that our findings are not accidental, but reveal some inherent nature of interpersonal communication.

## Discussion

In the present study, we used a fNIRS-based hyperscanning technic and analysis including WTC, sliding windows and k-mean clustering to obtain a set of representative dIBS states during different group communication. By calculating the parameters and temporal occurrence of the inter-brain states, we found that dense efficient interbrain states and sparse inefficient interbrain states appeared alternately and periodically, and the occurrence of efficient interbrain states was positively correlated with collaborative behaviors and group performance. Moreover, compared to the common communication, the occurrence of efficient interbrain states and state transitions were significantly higher during the creative communication, indicating a more active and intertwined neural network. These findings captured the variability and flexibility of inter-brain networks during group communication, and revealed the relationship between distinct dynamic IBS states and specific social behaviors, all of which contributing to our understanding of the multi-brain neural mechanisms involved in social communication.

Specifically, we clustered the dIBS of the AU and OC tasks and found three representative dIBS states. Results showed that State 1 had significantly higher globE and lower Lp than State 2 and State 3, and State 2 had significantly higher globE and lower Lp than State 3 (Figure 3B), which means that compared to State 3, State 1 and State 2 may be more beneficial for our interpersonal communication, especially State 1. During State 1, our brains are more aligned, more intertwined, and form a more efficient inter-brain network. The observed positive correlations between the occurrence rate of State 1 and group originality, as well as index of cooperation also support our explanation. Moreover, we found that during both creative and non-creative communication, dense efficient interbrain states and sparse inefficient interbrain states appeared periodically and alternately (Figure 3C). This may be related to spontaneous attentional fluctuations. Previous studies found that the electrophysiological activity of antagonistic, attention-relevant brain networks changes at the millisecond level and coordinates with moment-to-moment behavioral variability, which indicates that human attention is inherently dynamic and continuously shifting between external stimuli and internal thoughts (Kucyi et al., 2017, 2020). Therefore, it is difficult to maintain a longish state where we are highly focused and concentrated on the perspectives of others and efficiently absorb others’ ideas. In addition, results showed that in both AUT and OCT, State 1 occurs significantly less than State 2 and 3, and State 2 occurs significantly less than State 3. It may indicate the difficulty of entering into a state where group members experience productive interbrain information transmission during both creative and non-creative social communication. These findings uncovered the connections between dIBS states and social behaviors and revealed the dynamic nature of social communication.

To further explore the difference between the creative and non-creative group communication, we compared the occurrence rate of three dIBS states and the number of state transitions between AUT and OCT. AUT requires participants to report as many unusual and original uses as possible for an ordinary item. It is a classical divergent thinking task that is widely used in behavioral and neuroscience studies (Runco and Mraz, 1992; Fink et al., 2009). As for OCT, it requires participants to generate as many relevant characteristics as possible of a common item. In this regard, OCT often serves as a memory-retrieval task and is used as a control task for AUT (Fink et al., 2009; Chen et al., 2020). In the current study, results showed that during the AUT, the occurrence rate of States 1 and 2 was significantly higher than that during the OCT, and the occurrence rate of State 3 was significantly lower than that during the OCT. As mentioned above, States 1 and 2 contributed more to interpersonal communication and were associated with a higher level of mutual understanding as well as collaborative behaviors. Therefore, the higher occurrence rate of State 1 and State 2 during AUT may indicate that compared to the non-creative communication, participants paid more attention to each other, deeply evaluated and incorporated others’ ideas, and built more efficient inter-brain networks during the creative communication. Besides, the state transitions were significantly higher in the AUT than in the OCT, which suggests that participants’ dynamic inter-brain networks were more flexible, more active during creative communication. In a recent study, Li et al. (2021) clustered participants’ dIBS states during a product design task and a common model building task separately, and qualitatively compared these two tasks. They found that compared to the model building task, participants exhibited more complex and stronger IBS during the product design task. Here we expanded their findings into verbal communication tasks and quantitatively compared the properties of the dIBS states between creative and non-creative communication. With more detailed behavioral indices, we also observed a correspondence relationship between dIBS states and specific communication behaviors. During AUT, the group originality and cooperation behaviors were significantly positively correlated with the occurrence of State 1, which suggests that our social behaviors and dynamic inter-brain networks are harmoniously connected. Previous studies which focused on the static IBS networks also found stronger IBS during creative group collaborations (Lu et al., 2019; Mayseless et al., 2019). For instance, researchers found higher IBS in the r-DLPFC and r-TPJ during the creative cooperation tasks than the common cooperation tasks, and the increased IBS in these areas was positively correlated with behavioral indices of cooperation (Lu et al., 2019). Our findings are consistent with previous studies and provide a dynamic perspective to explore the unique inter-brain mechanisms of creative communication.

The sliding window approach is a common and useful method for dynamic FC analysis. It has been used to monitor temporal changes of the brain activity and even to classify and predict brain disorders (Du et al., 2018; Vidaurre et al., 2018). However, there is still a lack of a “gold standard” for determining the optimal window length (Shakil et al., 2016). Therefore, to examine the impact of different window lengths on the dIBS states, we used 5, 8, and 10 s as the length of sliding windows, separately. These window lengths were chosen based on our FOI (0.10–0.19 Hz, corresponding to the period between 5.2 and 10.5 s). As shown in Supplementary Figure 1, the dIBS patterns were generally consistent across different window lengths, which validates our findings.

In the present study, we analyzed the temporal variability and flexibility of the dynamic inter-brain networks during both creative communication and non-creative communication and found distinct dIBS states appeared periodically, and the increased efficient dIBS states were positively correlated with group performance, all of which suggest that there is a sophisticated correspondence between dIBS states and social behaviors. Moreover, creative communication has its unique characteristics. During creative communication, participants showed more efficient dIBS states and state transitions, which may indicate a higher level of comprehending and evaluating others’ ideas, building shared conceptional model, and collaboratively producing novel products.

There were still several limitations in this study. First, although AUT and OCT are classical cognitive tasks that are widely used in behavioral and neuroscience studies (Guilford, 1967; Fink et al., 2009; Hao et al., 2017), they are not natural social interactions. It should be cautious when generalizing these findings into actual social communication. Future research could use more varied and natural tasks to explore the variability of dynamic inter-brain networks during social communication. Moreover, since we set a time limit for the communication tasks (5 min for each task), participants may not be able to fully express their views during the communication. Future research could use more lenient time settings and explore the elaboration index of creative communication. Besides, we only recorded the activity of several brain areas such as the PFC and r-TPJ in this study. Future research could expand the coverage of the fNIRS optode probe sets so that the underlying inter-brain neural interactions can be fully explored.

## Data Availability Statement

The raw data supporting the conclusions of this article will be made available by the authors, without undue reservation.

## Ethics Statement

The studies involving human participants were reviewed and approved by the University Committee on Human Research Protection of East China Normal University. The patients/participants provided their written informed consent to participate in this study.

## Author Contributions

XW and NH conceived of the project and designed the experiments. XW and YH implemented the experiments and collected the data. XW, YZ, KL, and NH wrote the manuscript. All authors pre-processed the data, performed analyses, and discussed results.

## Conflict of Interest

The authors declare that the research was conducted in the absence of any commercial or financial relationships that could be construed as a potential conflict of interest.

## Publisher’s Note

All claims expressed in this article are solely those of the authors and do not necessarily represent those of their affiliated organizations, or those of the publisher, the editors and the reviewers. Any product that may be evaluated in this article, or claim that may be made by its manufacturer, is not guaranteed or endorsed by the publisher.
