# Supplementary figures and images for: Dynamic Inter-Brain Networks Correspond With Specific Communication Behaviors: Using Functional Near-Infrared Spectroscopy Hyperscanning During Creative and Non-creative Communication
Source: Front Hum Neurosci. 2022 Jun 2;16:907332. doi: 10.3389/fnhum.2022.907332 (PMC9201441; doi:10.3389/fnhum.2022.907332)

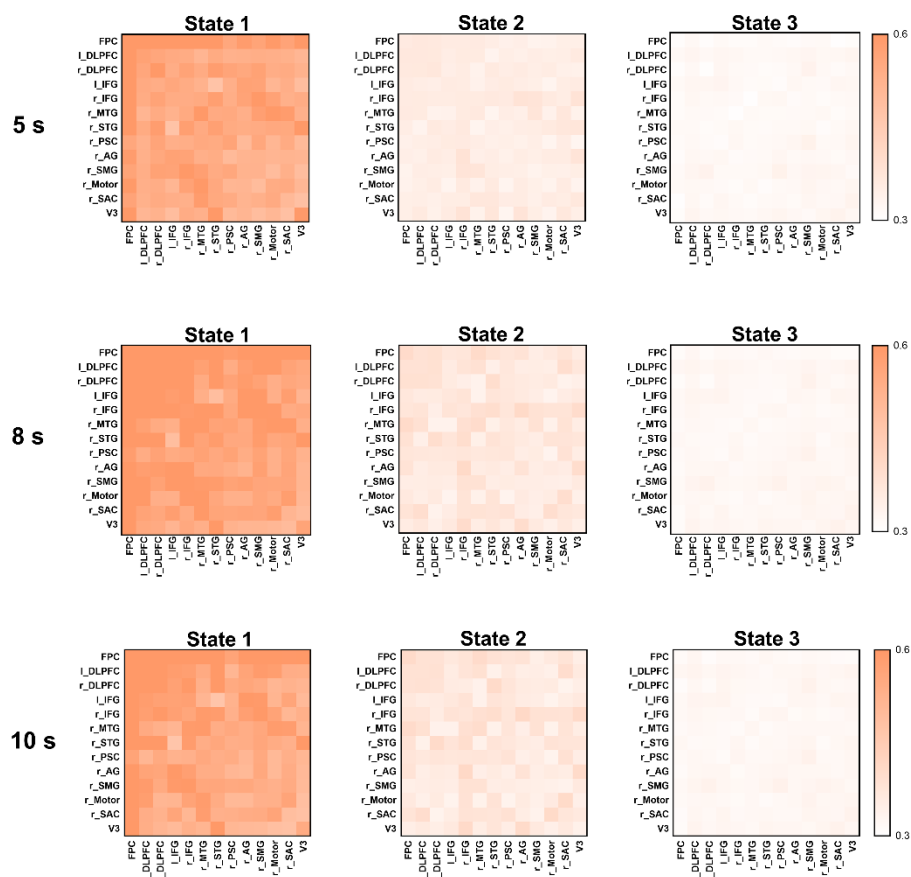

**Fig. S1. The effects of different window lengths (5s, 8s, and 10s).**

Supplement: Supplementary file 1 [file Image_1.pdf]
