# Supplementary material for: Dynamic Inter-Brain Networks Correspond With Specific Communication Behaviors: Using Functional Near-Infrared Spectroscopy Hyperscanning During Creative and Non-creative Communication
Source: Front Hum Neurosci. 2022 Jun 2;16:907332. doi: 10.3389/fnhum.2022.907332 (PMC9201441; doi:10.3389/fnhum.2022.907332)
Supplement: Supplementary file 2 [file Table_1.DOCX]

Table S1. The MNI Coordinates of each channel in a typical participant.

| **Channel** | **MNI coordinates (mm)** | | | **Brodmann Areas (Percentage)** |
| --- | --- | --- | --- | --- |
|  | **x** | **y** | **z** |  |
| **The 3*5 patch (PFC)** | | | | |
| 1 | *-40* | *61* | *0* | 10-Frontopolar area (63%) |
| 2 | *-16* | *73* | *5* | 10-Frontopolar area (84%) |
| 3 | *18* | *73* | *4* | 10-Frontopolar area (84%) |
| 4 | *41* | *63* | *1* | 10-Frontopolar area (69%) |
| 5 | *-48* | *49* | *8* | 46-Dorsolateral prefrontal cortex (71%) |
| 6 | *-28* | *66* | *15* | 10-Frontopolar area (87%) |
| 7 | *7* | *71* | *18* | 10-Frontopolar area (100%) |
| 8 | *30* | *67* | *14* | 10-Frontopolar area (96%) |
| 9 | *49* | *52* | *9* | 46-Dorsolateral prefrontal cortex (86%) |
| 10 | *-41* | *53* | *24* | 46-Dorsolateral prefrontal cortex (82%) |
| 11 | *-14* | *65* | *29* | 10-Frontopolar area (82%) |
| 12 | *16* | *67* | *28* | 10-Frontopolar area (91%) |
| 13 | *42* | *56* | *23* | 46-Dorsolateral prefrontal cortex (88%) |
| 14 | *-47* | *35* | *33* | 45-Pars triangularis Broca's area (76%) |
| 15 | *-26* | *53* | *36* | 46-Dorsolateral prefrontal cortex (51%) |
| 16 | *3* | *58* | *39* | 9-Dorsolateral prefrontal cortex (78%) |
| 17 | *30* | *54* | *34* | 46-Dorsolateral prefrontal cortex (62%) |
| 18 | *50* | *38* | *30* | 45-Pars triangularis Broca's area (88%) |
| 19 | *-35* | *36* | *46* | 9-Dorsolateral prefrontal cortex (91%) |
| 20 | *-11* | *50* | *49* | 9-Dorsolateral prefrontal cortex (98%) |
| 21 | *16* | *51* | *48* | 9-Dorsolateral prefrontal cortex (100%) |
| 22 | *39* | *39* | *43* | 9-Dorsolateral prefrontal cortex (78%) |
| **The 4*4 patch (****Right** **temporal-parietal-occipital cortex)** | | | | |
| 23 | *73* | *-25* | *-5* | 21-Middle temporal gyrus (96%) |
| 24 | *69* | *-50* | *0* | 37-Fusiform gyrus (46%) |
| 25 | *53* | *-78* | *-5* | 19-V3 (83%) |
| 26 | *71* | *-13* | *8* | 22-Superior temporal gyrus (92%) |
| 27 | *71* | *-39* | *12* | 22-Superior temporal gyrus (98%) |
| 28 | *62* | *-62* | *14* | 37-Fusiform gyrus (54%) |
| 29 | *46* | *-87* | *6* | 19-V3 (76%) |
| 30 | *70* | *-23* | *26* | 2-Primary somatosensory cortex (68%) |
| 31 | *66* | *-50* | *27* | 22-Superior temporal gyrus (61%) |
| 32 | *53* | *-75* | *24* | 39-Angular gyrus (84%) |
| 33 | *68* | *-6* | *32* | 43-Subcentral area (72%) |
| 34 | *68* | *-34* | *40* | 40-Supramarginal gyrus (73%) |
| 35 | *59* | *-60* | *38* | 39-Angular gyrus (84%) |
| 36 | *43* | *-81* | *35* | 19-V3 (70%) |
| 37 | *65* | *-20* | *46* | 1-Primary somatosensory cortex (57%) |
| 38 | *61* | *-43* | *50* | 40-Supramarginal gyrus (100%) |
| 39 | *48* | *-68* | *49* | 39-Angular gyrus (82%) |
| 40 | *57* | *-2* | *51* | 6-Pre-motor cortex (86%) |
| 41 | *60* | *-29* | *54* | 1-Primary somatosensory cortex (50%) |
| 42 | *51* | *-54* | *56* | 40-Supramarginal gyrus (84%) |
| 43 | *31* | *-70* | *56* | 7-Somatosensory association cortex (100%) |
| 44 | *52* | *-11* | *59* | 4-Pre-motor cortex (54%) |
| 45 | *45* | *-34* | *66* | 3-Primary somatosensory cortex (42%) |
| 46 | *32* | *-59* | *68* | 7-Somatosensory association cortex (96%) |
